# Synchronous Sternal Metastasectomy and Total Thyroidectomy for Differentiated Thyroid Cancer: A Rare Case Report

**DOI:** 10.7759/cureus.31294

**Published:** 2022-11-09

**Authors:** Anna Paspala, Dimitrios Papakonstantinou, Emmanouil Pikoulis, Periklis Tomos, Constantinos Nastos

**Affiliations:** 1 Third Department of Surgery, National and Kapodistrian University of Athens, Athens, GRC; 2 Third Department of Surgery, Attikon University Hospital, National and Kapodistrian University of Athens, Athens, GRC; 3 Thoracic Surgery, Attikon University Hospital, National and Kapodistrian University of Athens, Athens, GRC

**Keywords:** rare, management, survival, sternum, reconstruction, bone metastases, differentiated thyroid carcinoma

## Abstract

Bone metastases from thyroid cancer are mainly rare, while sternal metastases are extremely uncommon. Bone metastases might be either synchronous or metachronous to primary thyroid cancer. A 60-year-old male patient presented to our department with a painful, fixed and firm sternal mass. Preoperative imaging studies, such as neck ultrasound (US) and computed tomography (CT) of the chest, revealed a 6.5 cm nodule of the right thyroid lobe with high-risk malignancy characteristics and a massive metastatic mass of the anterior mediastinal, which was extended from the sternal notch to the third^ ^intercostal space. The diagnosis of papillary thyroid carcinoma with sternal metastatic lesions was established. After meticulous discussion in the multidisciplinary board of our hospital, a total thyroidectomy plus en-bloc resection of this massive sternal metastasis and adjuvant radioiodine therapy were decided. Eight months postoperatively, no recurrence has occurred in this patient. R0 resection of isolated bone metastasis of thyroid origin is still an optimal therapeutic decision for these patients. In cases of sternal metastasis, radical surgical resection with negative margins, including both resection of the lesion and reconstruction of the chest wall, in order to successfully maintain the chest wall's stability, is recommended.

## Introduction

Differentiated thyroid cancer (DTC) mainly metastasizes in locoregional lymph nodes and rarely to distant organs, such as the bone and lung. More specifically, papillary thyroid cancer (PTC) commonly metastasizes via the lymphatic pathway, whereas follicular thyroid neoplasms through vascular channels. Compared with PTC, patients with follicular thyroid cancer (FTC) have a higher risk of developing distant metastases and a higher proportion of cancer-related deaths [1]. The incidence of distant metastasis after the initial treatment of DTC is between 7% and 23%, while the frequency of patients diagnosed with DTC presenting with distant metastatic disease as the initial manifestation of cancer ranges from 1% to 9% [2,3].

Bone metastatic tumors from thyroid cancer are diagnosed in 4%-23% of patients, while sternal metastases are extremely uncommon, and they could be either synchronous or metachronous to primary thyroid cancer [4]. Bone metastases are associated with poorer survival outcomes in comparison to those with metastases to cervical lymph nodes [5,6]. Although radioactive iodine (RAI) ablation is the treatment of choice for distant metastases, bone metastases are not characterized by iodine avidity [5,6]. Therefore, surgical resection of resectable tumors could be a potential treatment strategy with better oncological outcomes [4].

Here, we present a case of papillary thyroid cancer with synchronous sternal metastasis that was treated with radical resection of both the primary and metastatic tumors at the same operation.

## Case presentation

**Figure 1:**
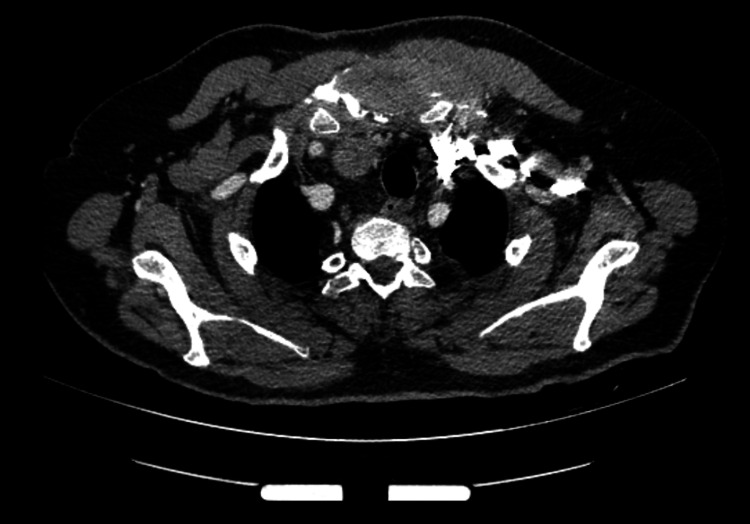
CT revealed a massive 4.7 × 4.6 × 4.5 cm sternal mass. CT: computed tomography.

A 60-year-old male patient presented to our department with a painful, fixed and firm sternal mass. Three months earlier, he underwent a T4-T5 discectomy and an en-bloc resection of a symptomatic metastatic lesion on T4, which was the first sign of a papillary thyroid carcinoma at the time. Histological examination revealed a metastasis from papillary thyroid carcinoma. Computed tomography (CT) of the chest demonstrated a 4.7 × 4.6 × 4.5 cm soft tissue mass originating and invading the sternum with a close relationship to the thyroid gland (Figure 1).

**Figure 2:**
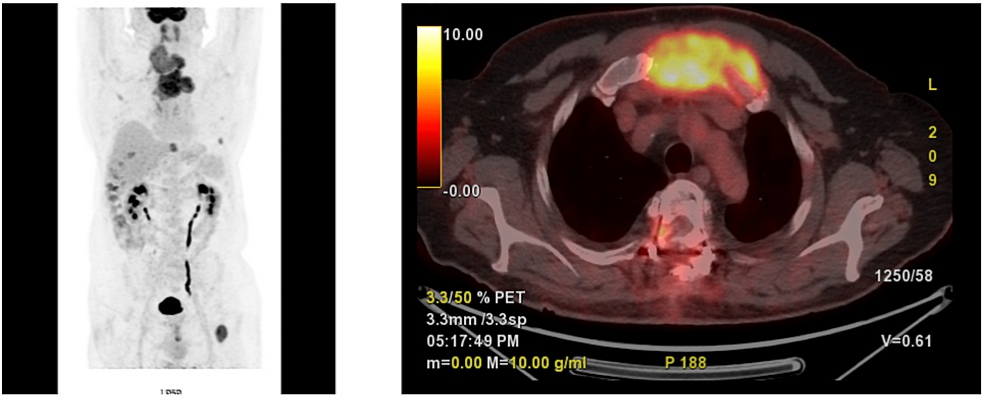
FDG-PET CT showed the sternal metastasis, which extended from the sternal notch to the third intercostal space. FDG: fluorodeoxyglucose, PET: positron emission tomography, CT: computed tomography.

Furthermore, a notably large nodule of the right thyroid lobe with an additionally marked deviation of the trachea. Neck ultrasound (US) revealed a 6.5-cm high-risk malignancy nodule of the right thyroid lobe (TIRADS 5) and a massive painful metastasis of the sternum. Fine-needle aspiration of the suspicious thyroid nodule established the diagnosis of DTC, excluding the presence of anaplastic thyroid cancer. His past medical and family history was unremarkable. Positron emission tomography (PET) CT showed the large thyroid neoplasm of the right thyroid lobe and an anterior mediastinal mass that was extended from the sternal notch to the third intercostal space (Figure 2).

After meticulous evaluation by a multidisciplinary team including endocrinologists, oncologists, and thoracic and endocrine surgeons, a total thyroidectomy plus en-bloc resection of sternal metastasis and adjuvant radioiodine therapy was decided. No lymph node metastases were present in the preoperative evaluation, and no lymph node dissection was performed. The aim of the resection was disease control and the treatment of severe pain during respiratory movements.

**Figure 3:**
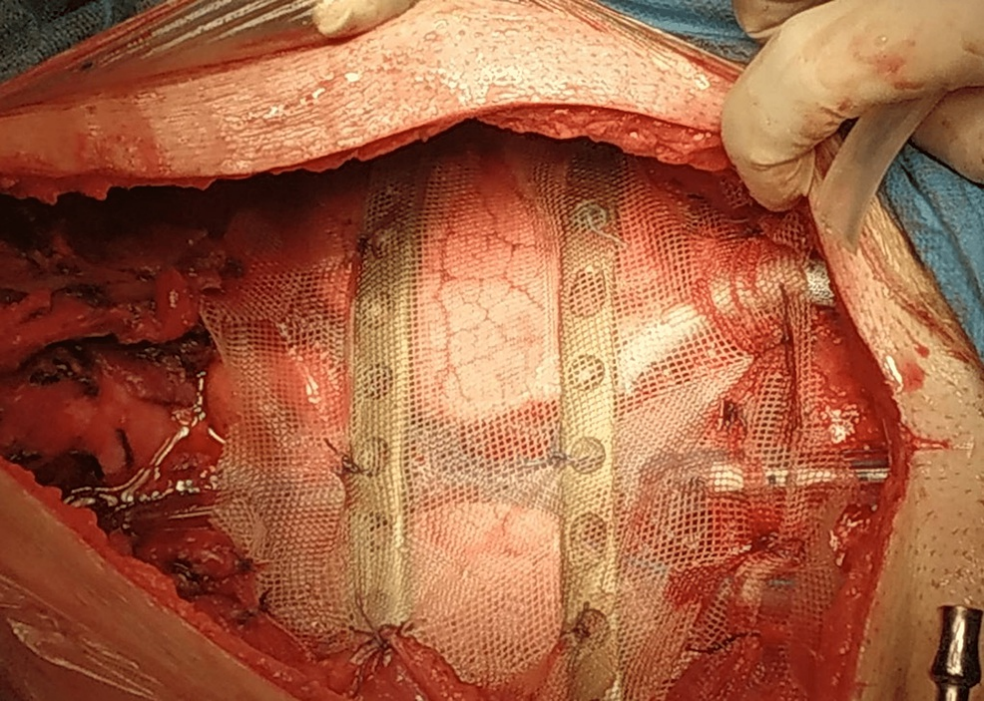
After meticulous en-bloc resection of the sternum and thyroid gland, the chest defect was reconstructed by using titanium bars and polypropylene mesh.

Upon operation, a 20-cm vertical incision was performed from the cricothyroid cartilage to the end of the sternal lesion. First of all, total thyroidectomy with identification and preservation of both recurrent laryngeal nerves and both external branches of the superior laryngeal nerves were completed by endocrine surgeons. Moreover, exposure of the sternal mass was started by the thoracic surgeons’ team. More precisely, a meticulous mobilization of the sternal lesion was performed by dissecting and excising the manubrium, the first, second, and third costal cartilages of the chest wall, plus the medial end of the clavicles bilaterally. The sternum was cut horizontally at the level of the third costochondral junction and the third ribs. The chest defect was reconstructed at the surgery by using titanium bars and polypropylene mesh as coverage (Figure 3).

The patient's postoperative course was complicated by a mild infection of the upper respiratory tract, which was successfully treated conservatively with intravenous administration of antibiotics. The rest of his postoperative course was uneventful, and he was discharged on the 13th postoperative day. Histopathological examination of the resected specimen revealed the presence of the infiltrative subtype of the follicular variant of PTC in both thyroid lobes, infiltrating the thyroid capsule in the right side with capsular invasion. Similarly, the same type of neoplasm was detected in the sternal metastasis. Eight months postoperatively, the patient remains disease-free and in excellent clinical condition after significant doses of radioactive iodine (RAI) ablation.

## Discussion

Distant metastases from differentiated thyroid neoplasms are described in approximately 25% of patients [2]. The most frequent sites of distant metastases are the lung and bone, accounting for about 50% and 18% of total cases, respectively [7]. The proportion of bone metastases in primary DTC ranges from 2% to almost 15%, whereas sternal metastases have been described in approximately 19% of patients with bone metastases from DTC [2]. Despite the fact that the 10-year survival rate of patients diagnosed with DTC is almost 98%, in cases with bone metastases, this rate ranges from 2% to 15% [5].

Several theories have been proposed in the current literature, regarding the mechanism and the pathways of bone secondary lesions spreading to the skeletous [8]. However, the main pathway of spreading is considered to be venous circulation and, more precisely, through Batson’s valveless plexus [9]. Batson’s plexus is connected with the brachiocephalic and superior vena cava, in which the inferior thyroid vein is drained [9]. Moreover, Batson’s plexus is located from the pelvis to the bones and drains the blood via the epidural and perivertebral veins [10]. On the other hand, other authors have suggested that malignant lesions are metastasized to the axial skeleton's red marrow because the blood flow is the highest [5]. Furthermore, arterial, and direct pathways of spread have a less favorable role, while lymphatic spread is a subject of debate in many studies [8,11].

The primary clinical manifestations of sternal metastases of thyroid neoplasms are pain and fractures [5]. The correlation of clinical suspicion with the appropriate imaging modalities can establish the diagnosis of these rare metastatic lesions [5]. US- or CT-guided biopsies may also be performed. Therefore, concerning the anatomical characteristics of these lesions such as the relation with the locoregional structures and their extension, the most useful imaging modalities are whole-body nonenhanced CT or magnetic resonance imaging (MRI) [5]. On the other hand, outcomes from functional imaging such as bone scintigraphy might not be reliable, as these imaging modalities are associated with common false-positive and false-negative results [5]. However, while the iodine whole-body scan has a high percentage of specificity and sensitivity, it is only effective for DTCs [12]. Regarding combined thyroglobulin-positive and iodine-negative cases, single photon emission CT and PET-CT are more efficient as diagnostic tools [12].

Due to the small number of published cases in the current literature, and the lack of randomized studies, there is no consensus and guidelines regarding the management of sternal metastases. However, according to several published studies, R0 resection of isolated bone metastasis of thyroid origin is still an optimal therapeutic decision for these patients. In cases of sternal metastasis, R0 resection, including both radical resection of the lesion and the reconstruction of the chest wall, in order to successfully maintain the chest's stability and prevent the paradoxical motion of the chest, is recommended. The surgical approach to chest wall reconstruction might include either prosthetic material, such as titanium bars and synthetic mesh, or myocutaneous flaps [13]. Unresectable tumors might receive efficient doses of RAI by performing a palliative cytoreductive surgery of the malignant burden. In the study conducted by Haugen et al., surgical resection and RAI for bone metastases in DTC are independently associated with a better prognosis, achieving 86.5% and 57.9% in 5- and 10-year survival rates, respectively [14].

In terms of long-term oncological outcomes for patients who underwent radical resection of bone metastases from DTC, in the multicentric retrospective study conducted by Fragnaud et al., overall survival for patients who underwent wide resection at 1, 5, 10, and 15 years was 76.2%, 63.6%, 63.6%, and 31.8%, respectively [15]. This study described much higher overall survival rates in comparison to the studies of Nakayama et al. and Satcher et al [16,17]. Moreover, Fragnaud et al. demonstrated that R0 and R1 resections were associated with a better prognosis compared to R2 resection, whereas the overall survival was extremely higher (82.3% vs 0%) when a resection of isolated bone metastasis was performed in comparison to patients with multi-metastatic bone lesions [15].

In addition to radical surgery and radioactive iodine (RAI), few other adjuvant therapies have been proposed for sternal and, in general, bone metastases from DTC. Concerning RAI-resistant metastatic lesions, chemotherapy unfortunately does not offer beneficial outcomes [18]. Furthermore, for unresectable bone metastases from DTC or when R0 resection could not be achieved, external beam radiotherapy might be an additional modality for treating these patients [19]. Nar Demirer et al. described a case of unresectable sternal metastasis from follicular thyroid carcinoma where external beam radiation therapy (EBRT) was used as a palliative method in order to decrease the tumor’s size and the symptoms that were associated with metastatic lesions [18].

## Conclusions

In conclusion, although metastatic lesions of the sternum in DTC are a rare entity, several imaging modalities are reliable in establishing a precise diagnosis. In our case, CT and PET-CT provided all the necessary data in order to design the appropriate therapeutic plan for our patient. When surgical resection is feasible and safe, it might be the best approach not only in terms of palliation of symptoms but also for oncological outcomes for patients with metastatic thyroid neoplasms.

## References

[1] Gilliland FD, Hunt WC, Morris DM, Key CR (1997). Prognostic factors for thyroid carcinoma. A population-based study of 15,698 cases from the surveillance, epidemiology and end results (SEER) program 1973-1991. Cancer.

[2] Osorio M, Moubayed SP, Su H, Urken ML (2017). Systematic review of site distribution of bone metastases in differentiated thyroid cancer. Head Neck.

[3] Sampson E, Brierley JD, Le LW, Rotstein L, Tsang RW (2007). Clinical management and outcome of papillary and follicular (differentiated) thyroid cancer presenting with distant metastasis at diagnosis. Cancer.

[4] Zettinig G, Fueger BJ, Passler C, Kaserer K, Pirich C, Dudczak R, Niederle B (2002). Long-term follow-up of patients with bone metastases from differentiated thyroid carcinoma-surgery or conventional therapy?. Clin Endocrinol (Oxf).

[5] Muresan MM, Olivier P, Leclère J (2008). Bone metastases from differentiated thyroid carcinoma. Endocr Relat Cancer.

[6] Durante C, Haddy N, Baudin E (2006). Long-term outcome of 444 patients with distant metastases from papillary and follicular thyroid carcinoma: benefits and limits of radioiodine therapy. J Clin Endocrinol Metab.

[7] Mizukami Y, Michigishi T, Nonomura A (1990). Distant metastases in differentiated thyroid carcinomas: a clinical and pathologic study. Hum Pathol.

[8] Maccauro G, Spinelli MS, Mauro S, Perisano C, Graci C, Rosa MA (2011). Physiopathology of spine metastasis. Int J Surg Oncol.

[9] Nathoo N, Caris EC, Wiener JA, Mendel E (2011). History of the vertebral venous plexus and the significant contributions of Breschet and Batson. Neurosurgery.

[10] Coleman RE (2006). Clinical features of metastatic bone disease and risk of skeletal morbidity. Clin Cancer Res.

[11] Edwards JR, Williams K, Kindblom LG (2008). Lymphatics and bone. Hum Pathol.

[12] Chua S, Gnanasegaran G, Cook GJ (2009). Miscellaneous cancers (lung, thyroid, renal cancer, myeloma, and neuroendocrine tumors): role of SPECT and PET in imaging bone metastases. Semin Nucl Med.

[13] Lequaglie C, Massone PB, Giudice G, Conti B (2002). Gold standard for sternectomies and plastic reconstructions after resections for primary or secondary sternal neoplasms. Ann Surg Oncol.

[14] Haugen BR, Alexander EK, Bible KC (2016). 2015 American Thyroid Association Management Guidelines for Adult Patients with Thyroid Nodules and Differentiated Thyroid Cancer: The American Thyroid Association Guidelines Task Force on Thyroid Nodules and Differentiated Thyroid Cancer. Thyroid.

[15] Fragnaud H, Mattei JC, Le Nail LR (2022). Mid and long-term overall survival after carcinologic resections of thyroid cancer bone metastases. Front Surg.

[16] Nakayama R, Horiuchi K, Susa M (2014). Clinical outcome after bone metastasis (BM) surgery in patients with differentiated thyroid carcinoma (DTC): a retrospective study of 40 cases. Jpn J Clin Oncol.

[17] Satcher RL, Lin P, Harun N, Feng L, Moon BS, Lewis VO (2012). Surgical management of appendicular skeletal metastases in thyroid carcinoma. Int J Surg Oncol.

[18] Nar Demirer A, Ayturk S, Tutuncu NB, Gursoy A, Pak Y, Demirag NG (2009). Unresectable huge sternal and mediastinal metastasis of follicular thyroid carcinoma; radiotherapy as first-line and palliative therapy. Exp Clin Endocrinol Diabetes.

[19] Brierley JD, Tsang RW External‐beam radiation therapy in the treatment of differentiated thyroid cancer. Seminars in Surgical Oncology.

